# Globaltest confidence regions and their application to ridge regression

**DOI:** 10.1002/bimj.202000063

**Published:** 2021-05-27

**Authors:** Ningning Xu, Aldo Solari, Jelle Goeman

**Affiliations:** ^1^ Department of Biomedical Data Sciences Leiden University Medical Center Leiden The Netherlands; ^2^ Department of Economics Management and Statistics University of Milano‐Bicocca Milano Italy

**Keywords:** confidence regions, high dimensional, tuning parameter selection

## Abstract

We construct confidence regions in high dimensions by inverting the globaltest statistics, and use them to choose the tuning parameter for penalized regression. The selected model corresponds to the point in the confidence region of the parameters that minimizes the penalty, making it the least complex model that still has acceptable fit according to the test that defines the confidence region. As the globaltest is particularly powerful in the presence of many weak predictors, it connects well to ridge regression, and we thus focus on ridge penalties in this paper. The confidence region method is quick to calculate, intuitive, and gives decent predictive potential. As a tuning parameter selection method it may even outperform classical methods such as cross‐validation in terms of mean squared error of prediction, especially when the signal is weak. We illustrate the method for linear models in simulation study and for Cox models in real gene expression data of breast cancer samples.

## 1 INTRODUCTION

Confidence regions play a fundamental role in statistical inference. Points within a confidence region can be viewed as reasonable candidates for the true parameter. By distinguishing between acceptable and unacceptable values, confidence regions can be used to select the tuning parameter of penalized regression models.

The rationale for the confidence region approach to tuning parameter selection is as follows. If a model is not in the confidence region, it has significantly worse fit than the true model. It makes sense, therefore, to restrict attention to only models inside the confidence region. In the context of penalized methods, among all acceptable models we may prefer the model with the smallest penalty rather than the midpoint of the confidence region. This is the least complex, and therefore hopefully least overfitting model among all acceptable models.

There have been many confidence region approaches proposed for tuning parameter selection, for example, Obenchain (1977), McCabe (1978), and Oman (1981) proposed to use classical *F*‐test to select the tuning parameter for ridge regression (Hoerl & Kennard, 1970). More recently, Gunes and Bondell (2012) applied the confidence region approach in variable selection with adaptive LASSO (Zou, 2006). A similar idea was proposed by Jiang et al. (2008) for model selection in linear mixed models. In one variant, their “fence” around all acceptable models is exactly the border of the likelihood ratio test confidence region. Within the fence they also select the model that minimizes the penalty. Note that the confidence regions used in these papers are all obtained by inverting the likelihood ratio test (i.e., the *F*‐test for linear models), which is only applicable for low‐dimensional data.

The purpose in this work is to extend the confidence region approach to high dimensions for a wide range of generalized linear models and Cox models, for which globaltest Goeman et al. (2004) can be used. We build confidence regions based on the globaltest. In the context of linear models, Goeman et al. (2006) showed that the globaltest is more powerful than the *F*‐test when large variance principal components of the design matrix explain more of the variance of the outcome than the small variance ones. More importantly, the globaltest is powerful in high dimensions, especially when there are many predictors with weak effects.

In many biological data examples, it is common that good predictive ability can be obtained from the cumulative effect of many weak predictors even though they might be too weak to be identifiable individually. This is the scenario where both ridge regression and globaltest work well. Therefore, we concentrate on the combination of globaltest and ridge regression in this paper, that is, using the confidence region of globaltest to choose the tuning parameter for ridge regression.

The confidence region approach is more attractive than other criteria, such as, classically, cross‐validation (CV) (Breiman & Spector, 1992) and information criteria, for several reasons. First, the confidence region approach can be viewed as a testimation procedure (Rahman & Gokhale, 1996), for which the resulting “testimator” is corresponding to the least overfitting estimator that is tested significant by globaltest at a prespecified significance level α. Ridge regression selects either the full model or the null model. When the null model is false, the probability of choosing the full model converges to 1 for a fixed alternative because the global test is consistent (Goeman et al., 2006). When the null model is true, the confidence region method can guarantee that the probability of selecting the null model is asymptotically 1−α. This can be an important property because it may prevent false predictive claims from entering the literature. Second, the significance level α can take the role of the classical tuning parameter λ, of which the scale is arbitrary, making it difficult to interpret. The α is well calibrated and, due to its direct interpretation as an error rate, may be chosen a priori at a reasonable level of acceptable type I error control. By linking tuning parameter selection to inferential theory in this way, tuning parameter selection becomes less of an algorithmic black box.

The classical choice of α=5% is sensible if stringent error rate control is crucial, but this will lead to conservative model fits. In prediction modeling, many methods used in practice have type I error rate of around 50% (Gunes & Bondell, 2012). In contexts where weak type I error rate control is more desirable, the confidence region approach at level of 50% can therefore be expected to produce results very close to those of classical methods such as CV but is much faster than CV. Type I error at level 50% has also been recommended by Aitkin (1974) to prevent the conservativeness of a simultaneous variable selection procedure.

The structure of the paper is as follows. We will describe the confidence region of globaltest and revisit ridge regression, and then present our method in a general way in Section 2. The properties of the globaltest and ridge estimator for linear models will be discussed in more detail in Section 3, in which we compare the globaltest confidence region with the *F*‐test confidence region. A numerical study compares our proposed method with other methods in Section 4, where we also perform a real data analysis based on three high‐dimensional breast cancer data sets.

## 2 THE GLOBALTEST CONFIDENCE REGION APPROACH

### 2.1 The globaltest

Suppose we have data with n observations and p predictors. X is an n×p design matrix whose columns correspond to p predictors. A regression model relates the response to the predictors through the linear predictors $x_{i}^{T}\beta$, where $x_{i}^{T}=\left(x_{i1},\ldots ,x_{ip}\right)$ is the ith row of X and $\beta ={\left(\beta_{1},\cdots ,\beta_{p}\right)}^{T}$ are the unknown model coefficients.

We assume a generalized linear model. Let $y={\left(y_{1},\ldots ,y_{n}\right)}^{T}$ be the vector of responses, where $y_{i}$ follows a distribution in the exponential family. The model assumes the mean of the response and the linear predictors are related by $g\left(E\left(y_{i}\right)\right)=x_{i}^{T}\beta$, where g is a monotone link function, for example, the identity function for the linear model or the logit function for the logistic model. Extensions to the Cox proportional hazard model are straightforward and we come to those in Section 5.

Suppose that we are interested in testing the following null hypothesis:

$$H_{0}:\beta =\beta_{0}$$

against the alternative $H_{1}:\beta \neq \beta_{0}$. Goeman et al. (2011) derived the following globaltest statistic:

$${\hat{S}}_{\beta_{0}}=s^{T}s-\text{trace}\left(I\right),$$

where $s=\frac{\partial \ell (\beta )}{\partial \beta }{|}_{\beta =\beta_{0}}$ is the score of β at $\beta_{0}$, ℓ(β) is the log‐likelihood of the model, and $I=-\frac{\partial^{2}\ell \left(\beta \right)}{\partial \beta \partial \beta^{T}}{|}_{\beta =\beta_{0}}$ is the observed information matrix. Because trace(I) does not depend on the response (Goeman et al., 2011), ${\hat{S}}_{\beta_{0}}$ is equivalent to

$$S_{\beta_{0}}=s^{T}s.$$

Then by inverting the statistic $S_{\beta_{0}}$ we get the 1−α confidence region of globaltest:

(1) $$C_{\alpha }^{\mathrm{gt}}=\left\{\beta_{0}\in R^{p}:S_{\beta_{0}}\leq c_{\alpha }\right\}.$$

Here $c_{\alpha }$ is the 1−α quantile of the null distribution of $S_{\beta_{0}}$. Goeman et al. (2011) derived the exact null distribution of $S_{\beta_{0}}$ for linear models and asymptotic null distribution for other generalized linear models using the algorithms developed by Imhof (1961) and Robbins and Pitman (1949). The implementation of the globaltest can be referenced to the R package globaltest (Goeman et al., 2010).

### 2.2 Ridge regression

Ridge regression, first proposed by Hoerl and Kennard (1970), is a useful technique for analyzing data that suffer from multicollinearity. In common with other shrinkage methods such as LASSO (Tibshirani, 1996) and the elastic net (Zou & Hastie, 2005), ridge regression aims at maximizing the likelihood function by adding a penalty to the model coefficients.

A general form for penalized regression is given by the following optimization problem:

$${\hat{\beta }}_{\lambda }=\underset{\beta \in R^{p}}{\mathrm{arg max}}\left\{\ell \left(\beta \right)-p_{\lambda }\left(\beta \right)\right\},$$

where $p_{\lambda }\left(\beta \right)=\lambda p\left(\beta \right)$ is the penalty term. For ridge regression, $p\left(\beta \right)={∥\beta ∥}_{2}^{2}$, where ${∥\cdot ∥}_{q}$ is the $L_{q}$ norm.

Therefore, ridge regression puts an additional penalty term on the parameters instead of just maximizing the log‐likelihood function, where the penalty term is the tuning parameter λ times the square of the $L_{2}$ norm of the coefficients vector β. In the extreme cases, when λ=0, the ridge estimator is simply the maximum likelihood estimation (MLE), while when λ approaches infinity, all the coefficients tend to zero. Consequently, the performance of ridge regression largely depends on the tuning parameter λ that balances the trade‐off between bias and variance.

Interest in the applications of ridge regression has increased as high‐dimensional data are increasingly common. Bøvelstad et al. (2007) compared several dimension reduction or parameter shrinkage methods for high‐dimensional data and concluded that ridge regression has the overall best prediction performance. Based on the ridge estimation, Bühlmann et al. (2013) proposed a method for constructing p‐values for general hypotheses in the high‐dimensional linear model. An automatic method was derived by Cule and De Iorio (2013) to choose the ridge parameter for high‐dimensional data. Van Wieringen and Peeters (2016) investigated the properties of the ridge estimation of the precision matrix for high‐dimensional data. Recently, ridge regression was applied to VAR(1) models by Miok et al. (2017). Ridge regression has a good reputation in prediction for high‐dimensional data.

### 2.3 Choice of the tuning parameter

The main idea of the confidence region approach is to choose the $L_{2}$‐sparsest solution ${\hat{\beta }}_{\lambda }$ contained in the confidence region for β. The solution is the first time that the path of ridge estimator starting from λ=∞ to λ=0 reaches the boundary of the confidence region. When the ridge path is completely included in the confidence region, the null model is chosen. Gunes and Bondell (2012) used the likelihood ratio test for low‐dimensional data. However, the high dimensionality renders this test inapplicable. We replace it with the globaltest in this paper.

As a consequence, the tuning parameter selected by the globaltest confidence region at level 1−α is

(2) $$\lambda^{\mathrm{gt}}\left(\alpha \right)=\sup\left\{\lambda \in \left[0,\infty \right):{\hat{\beta }}_{\lambda }\in C_{\alpha }^{\mathrm{gt}}\right\}.$$

Given a specific value of α, the solution for λ in (2) is fully determined by α, suggesting to use the penalized estimate ${\hat{\beta }}_{\lambda^{\mathrm{gt}}\left(\alpha \right)}$. Similarly, for a given tuning parameter λ, it can be checked whether ${\hat{\beta }}_{\lambda }$ lies in the 1−α confidence region $C_{\alpha }^{\mathrm{gt}}$, or which is the smallest level α such that ${\hat{\beta }}_{\lambda }\in C_{\alpha }^{\mathrm{gt}}$, that is,

$$\alpha^{\mathrm{gt}}\left(\lambda \right)=\inf\left\{\alpha \in \left[0,1\right]:{\hat{\beta }}_{\lambda }\in C_{\alpha }^{\mathrm{gt}}\right\}.$$

A mapping, therefore, can be built between the tuning parameter λ and the confidence level parameter α. Under the assumption that a smaller significance level α corresponds to a larger confidence region, we have that λ(α) is a nonincreasing function on α, or equivalently, α(λ) is a nonincreasing function on λ. Many of the commonly used tests satisfy this assumption, such as the likelihood ratio test, Wald test and globaltest: for $\alpha_{1}\leq \alpha_{2}$, $c_{\alpha_{1}}\geq c_{\alpha_{2}}$ holds so that $C_{\alpha_{1}}⊇C_{\alpha_{2}}$, thereby $\lambda \left(\alpha_{1}\right)\geq \lambda \left(\alpha_{2}\right)$.

## 3 LINEAR MODELS

For the specific case of linear models, we show more detail about the properties of the globaltest and the ridge estimator, and then compare the confidence regions of the globaltest and *F*‐test.

### 3.1 Detectable regions for the globaltest

Consider a linear model

$$y∼N(X\beta ,\sigma^{2}I_{n}),$$

where $I_{n}$ is the n×n identity matrix. Then the globaltest statistic for the linear model becomes

(3) $$S_{\beta_{0}}=\frac{∥X^{T}\left(y-X\beta_{0}\right){∥}_{2}^{2}}{∥y-X\beta_{0}{∥}_{2}^{2}}.$$

Goeman et al. (2006) proved that the globaltest is the locally most powerful test on average in a neighborhood of the null hypothesis. However, especially when p≫n, there are points $\beta \neq \beta_{0}$ for which the globaltest has negligible power. More specifically, let p>n and $X^{T}X=\sum_{i=1}^{n}\gamma_{i}V_{i}$, where $\gamma_{1}\geq \cdots \geq \gamma_{n}\geq 0$ are the nonzero eigenvalues of $X^{T}X$ and $V_{i}$ is the p×p projection matrix that projects onto the eigenvector of $X^{T}X$ corresponding to the eigenvalue $\gamma_{i}$. As detailed in Goeman et al. (2006), the globaltest is less powerful for the points whose expected test statistic under alternative hypothesis is smaller than that under the null hypothesis. The difference of the expectations under alternative and null hypotheses is approximately proportional to the covariance of $\gamma ={\left(\gamma_{1},\ldots ,\gamma_{n}\right)}^{T}$ and $r^{2}={\left(r_{1}^{2},\ldots ,r_{n}^{2}\right)}^{T}$, where

(4) $$r_{i}^{2}=\frac{\gamma_{i}{\left(\beta -\beta_{0}\right)}^{T}V_{i}\left(\beta -\beta_{0}\right)}{{\left(\beta -\beta_{0}\right)}^{T}X^{T}X\left(\beta -\beta_{0}\right)+n\sigma^{2}},$$

and $r^{2}=\sum_{i=1}^{n}r_{i}^{2}$ is the fraction of variance of y explained by the alternative hypothesis. Thus, the detectable region for the globaltest is defined as

$$D=\{\beta_{0}\in R^{p}:\mathrm{cov}\left(\gamma ,r^{2}\right)>0\}.$$

The globaltest has good power for testing the points inside D, as opposed to the points outside.

The ridge estimator for the linear model has the following form:

$${\hat{\beta }}_{\lambda }={\left(X^{T}X+\lambda I_{p}\right)}^{-1}X^{T}y.$$

Based on the singular value decomposition of $X=U\Gamma^{1/2}V^{T}$, ${\hat{\beta }}_{\lambda }$ can be written as

(5) $${\hat{\beta }}_{\lambda }=\sum_{i=1}^{n}\frac{\gamma_{i}^{1/2}}{\gamma_{i}+\lambda }v_{i}u_{i}^{T}y.$$

Here $v_{i}$ and $u_{i}$ are the ith columns of V and U, where $v_{i}$ is called the ith principal component direction of X.

It can be seen from (5) that $\frac{\gamma_{i}^{1/2}}{\gamma_{i}+\lambda }$ is an increasing function of $\gamma_{i}$ when $\lambda >\gamma_{i}$ and is a decreasing function of $\gamma_{i}$ when $\lambda <\gamma_{i}$. In other words, the ridge estimator ${\hat{\beta }}_{\lambda }$ will be more correlated with the large variance principal components of X than with those with small variance when $\lambda >\gamma_{1}$; it will be more correlated with the small variance principal components than with those with the large variance when $\lambda <\gamma_{\min(n,p)}$. Hence, the ridge path starting from λ=∞ to λ=0 would first move along the direction of strong principal components, and then change into the direction of small principal components until reaching the MLE.

**FIGURE 1:**
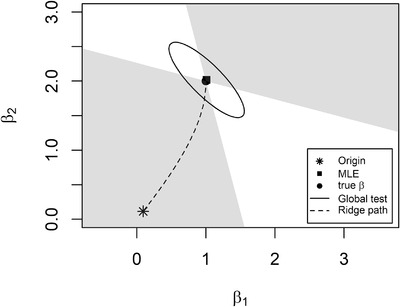
Detectable region (gray shaded area) of the globaltest *Note*: * denotes the origin of ridge path, and ▪ is the end point, MLE. • represents the true coefficients. The ridge path is represented by the dashed line, and the solid line indicates the boundary of the globaltest confidence region

Figure 1 illustrates the detectable region of the globaltest and the direction of ridge path for the Gaussian linear model with n=50 and p=2. The true coefficients are $\beta ={\left(1,2\right)}^{T}$, and the correlation between these two predictors is ρ=0.6. It can be seen that the ridge path approaches to the MLE first in the direction of the strong principal component of the design matrix, which is the direction of minor axis of the ellipse, and then turns into the direction of the weak principal component, which is the direction of major axis.

### 3.2 Comparisons with the Scheffé confidence region

The *F*‐test statistic for testing $H_{0}:\beta =\beta_{0}$ against $H_{1}:\beta \neq \beta_{0}$ is given by

$$T_{\beta_{0}}=\frac{∥X\hat{\beta }-X\beta_{0}{∥}_{2}^{2}/p}{{\hat{\sigma }}^{2}},$$

which follows an *F* distribution with degrees of freedom p and n−p under $H_{0}$, where ${\hat{\sigma }}^{2}={∥y-X\hat{\beta }∥}_{2}^{2}/\left(n-p\right)$ and $\hat{\beta }={\left(X^{T}X\right)}^{-1}X^{T}y$. The Scheffé confidence region obtained by inverting the *F*‐test statistic is a hyperellipsoid centered at the MLE:

$$C_{\alpha }^{\mathrm{ft}}=\left\{\beta_{0}\in R^{p}:{\left(\beta_{0}-\hat{\beta }\right)}^{T}X^{T}X\left(\beta_{0}-\hat{\beta }\right)\leq p{\hat{\sigma }}^{2}f_{p,n-p}^{\alpha }\right\},$$

where $f_{p,n-p}^{\alpha }$ is the 1−α quantile of the *F* distribution with p and n−p degrees of freedom.

It is interesting to note that the confidence region of the globaltest is not always ellipsoid. Theoretically, for a given confidence level 1−α, the border of the globaltest confidence region for linear models based on (1) and (3) is

$$\frac{{\left(y-X\beta_{0}\right)}^{T}XX^{T}\left(y-X\beta_{0}\right)}{{\left(y-X\beta_{0}\right)}^{T}\left(y-X\beta_{0}\right)}=c_{\alpha },$$

which is equivalent to

(6) $$\beta_{0}^{T}\left(X^{T}XX^{T}X-c_{\alpha }X^{T}X\right)\beta_{0}-2y^{T}\left(XX^{T}X-c_{\alpha }X\right)\beta_{0}+y^{T}\left(XX^{T}-c_{\alpha }I_{n}\right)y=0.$$

For p=2, Equation (6) is exactly the general form of a conic section. The type of the conic section can be determined by the sign of $\delta =det(X^{T}XX^{T}X-c_{\alpha }X^{T}X)$ (Desgraupes, 2013). Then one has the following classifications based on δ:

- if δ>0, (6) is an ellipse;
- if δ=0, (6) is a pair of parallel lines;
- if δ<0, (6) is a hyperbola.

​

**FIGURE 2:**
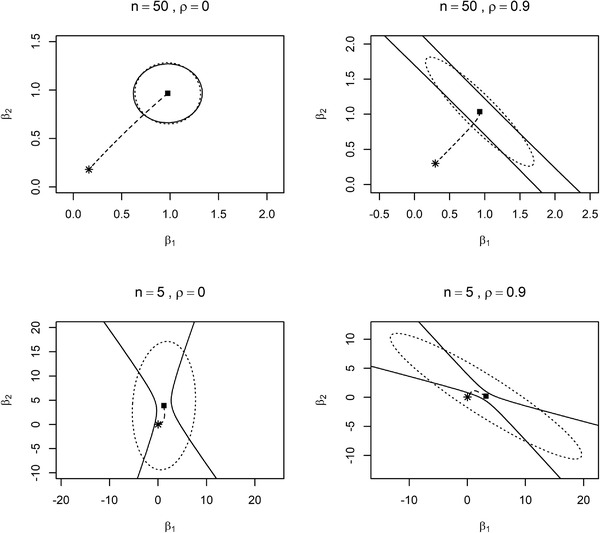
Confidence regions of the globaltest and *F*‐test for n=50,5,p=2 with correlations ρ=0,0.9 *Note*: The dashed line denotes the ridge path with * is the origin and ▪ is MLE. The solid line denotes the globaltest confidence region and the dotted line denotes the Scheffé confidence region

Figure 2 shows the comparisons of the confidence regions of the globaltest and *F*‐test for simulated data with n∈{5,50} samples and two predictors, for which the correlation is ρ∈{0,0.9}. It is shown that decreasing the sample size or increasing the correlation makes the confidence region of the globaltest become narrower than the Scheffé confidence region along the direction of the strong principal component. This results in smaller λ chosen by the confidence region of the globaltest than the *F*‐test provided that the ridge path comes from the detectable region of globaltest. Note that it can happen that the whole ridge path is completely included in the confidence region, as the example of n=5 in Figure 2 demonstrates. In that case λ=∞ is chosen.

## 4 SIMULATIONS

We conduct two simulations to illustrate the points raised in previous sections. One is to show the comparisons between the globaltest‐based method and the *F*‐based method. The other is to describe the predictive ability of the proposed method as compared with other methods, both for low‐ and high‐dimensional data. The design matrix X in the simulated data is based on a real gene expression data set from the breast cancer study published by Van't Veer et al. (2002) and Van De Vijver et al. (2002), including 14,318 gene features for 337 breast cancer patients (after removing the missing values). We take a low‐dimensional setting with the first n=300 patients and the first p=50 gene features considered for the first simulation so that *F*‐test can also be applied. A high‐dimensional setting is added in the second simulation with the first n=300 patients and the first p=1000 gene features. We use the linear model $y∼N(X\beta ,\sigma^{2}I)$ to generate the output variable. All simulation results shown below are based on 1000 replications.

### 4.1 Comparison with the *F*‐based method

We use the same setup of the true coefficients as in Goeman et al. (2006) so that we can gain insights into the properties of the globaltest. In terms of singular value decomposition, we have $X=U\Gamma^{1/2}V^{T}$ with U be an n×min(n,p) (semi)orthogonal matrix, V a p×min(n,p) (semi)orthogonal matrix and Γ is a min(n,p)×min(n,p) diagonal matrix. It is shown in Goeman et al. (2006) that globaltest is powerful especially when the large principal components explain more of the variance of the response than the small ones. We therefore define the true model coefficients β in a way that it can vary the amount of variance explained by the principal components by varying s:

(7) $$\beta =V\gamma^{s/2},$$

where γ are the nonzero diagonals of Γ.

When s>0, the large variance principal components will have large coefficients and also large $r^{2}$ for fixed $\sigma^{2}$, in terms of (7) and (4). Positive correlations between γ and $r^{2}$ are thus obtained, leading to good power of the globaltest. When −1<s<0, the large variance principal components will have smaller coefficients but larger $r^{2}$ than those small variance principal components. Although when s<−1, y is totally determined by the small variance principal components. Thus, when s becomes negative, globaltest tends to lose power due to the negative correlation between γ and $r^{2}$.

Given the value of $r^{2}$ and the true coefficients β, we can calculate $\sigma^{2}$ based on Equation (4). We then use linear regression to generate the response y. The larger $r^{2}$, the more variance of y explained by the true model. The larger s, the more powerful the globaltest. Goeman et al. (2006) argued that s>0 is fortunately common in the real data, for which the globaltest has good power. For negative s, globaltest has negligible power even for large $r^{2}$.

Table 1 shows the proportion of times that the tuning parameter selected by the globaltest‐based method is smaller than that by the *F*‐based method. It is shown that the globaltest‐based method would choose smaller, that is, less conservative, tuning parameters in comparison to the *F*‐based method for large values of s, because the power of the globaltest in this case is better than that of *F*‐test. For negative values of s, which cause negative correlations between γ and $r^{2}$, the *F*‐based method outperforms the globaltest‐based method. For example, the proportion is 0.946 for s=1.5 and $r^{2}=0.15$, whereas for s=−1.5 with the same $r^{2}$, the proportion is 0. This is consistent with the properties of the globaltest discussed in Goeman et al. (2006).

**TABLE 1:** Proportion of times that $\lambda^{\mathrm{gt}}<\lambda^{\mathrm{ft}}$, where $\lambda^{\mathrm{gt}}$ and $\lambda^{\mathrm{ft}}$ denote the tuning parameters selected by the globaltest‐based method and *F*‐based method, respectively, with confidence level 95%

	$r^{2}$			
s	0.02	0.05	0.1	0.15
1.5	0.733	0.907	0.935	0.946
1	0.715	0.902	0.926	0.936
0.5	0.694	0.869	0.888	0.881
0	0.629	0.739	0.692	0.594
−0.5	0.486	0.407	0.237	0.109
−1	0.373	0.131	0.042	0.008
−1.5	0.291	0.056	0.005	0.000

### 4.2 Predictive ability

To investigate the predictive ability of the confidence region method, we calculate the mean squared error (MSE) of the predictions in terms of

$$\mathrm{MSE}=\frac{1}{n}\sum_{i=1}^{n}{\left(x_{i}^{T}\beta -x_{i}^{T}{\hat{\beta }}_{\lambda }\right)}^{2},$$

where ${\hat{\beta }}_{\lambda }$ is the ridge estimate. For the low‐dimensional setting, we compare the globaltest‐based method with CV (5‐CV, LOOCV, and generalized CV (GCV), Golub et al., 1979), information criteria (AIC, Akaike, 1973; and its variant AICc, Cavanaugh et al., 1997; BIC, Schwarz et al., 1978; and its modified versions mBIC, mBIC2, Żak‐Szatkowska and Bogdan, 2011; GIC, Fan and Tang, 2013; and RIC, Foster and George, 1994) and the *F*‐based method.

**FIGURE 3:**
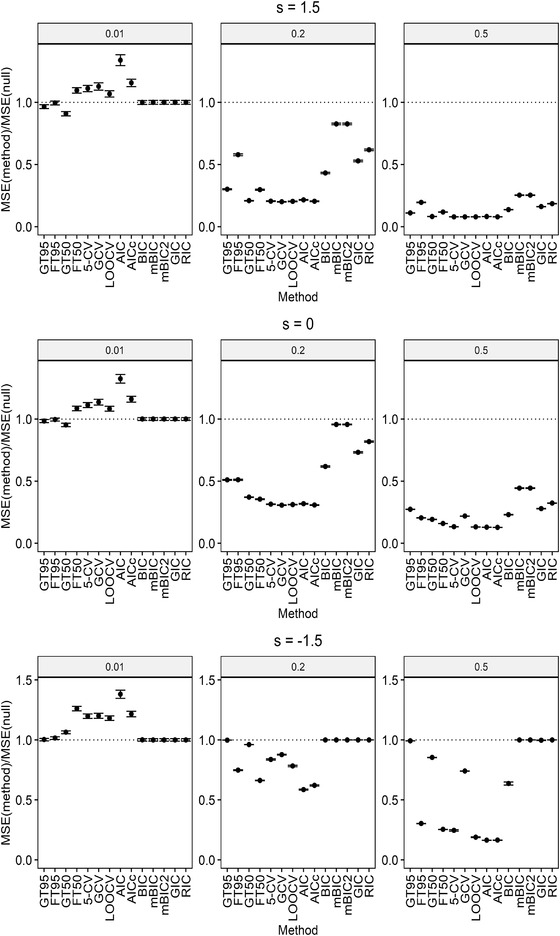
MSE relative to MSE of the null model: MSE(method)/MSE(null) ± standard errors in low dimensions for $r^{2}=0.01,0.2,0.$ *Note*: The dotted line corresponds to MSE of the null model

**FIGURE 4:**
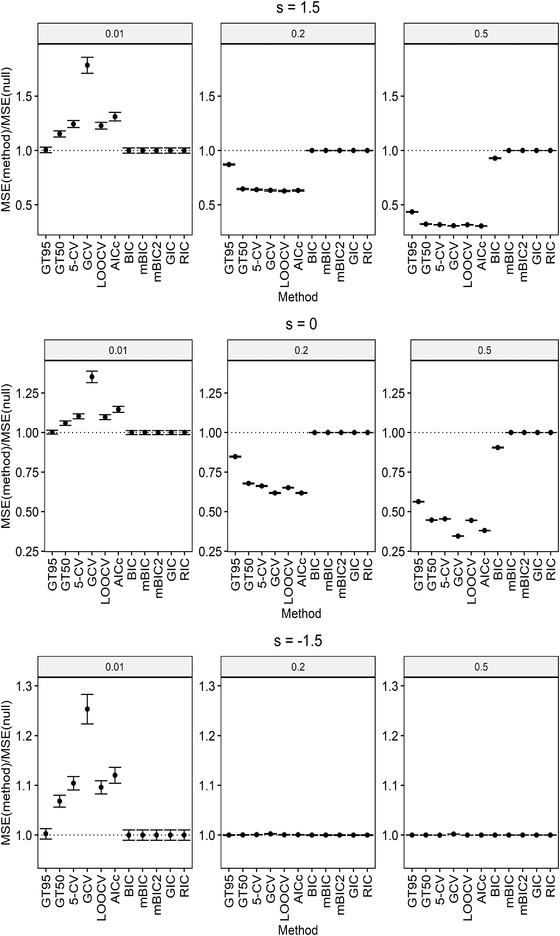
MSE relative to MSE of the null model: MSE(method)/MSE(null) ± standard errors in high dimensions for $r^{2}=0.01,0.2,0.5$ *Note*: The dotted line corresponds to MSE of the null model

The information criteria measure the balance between model fit and model complexity by minimizing the following expression (van Wieringen, 2020):

−2×ℓ(λ)+penaltyonmodelcomplexity,

where ℓ(λ) is the penalized log‐likelihood and $\mathrm{mc}=\sum_{i=1}^{\min(n,p)}\frac{\gamma_{i}}{\gamma_{i}+\lambda }$ denotes the model complexity for ridge regression. We use the R package penalized (Goeman, 2012) to calculate ℓ(λ). Penalties used in all of the information criteria mentioned above are summarized in Table 2. The CV results are also calculated from R package penalized. For the high‐dimensional setting, we exclude AIC and *F*‐based method, as they totally break down. The results for both low and high dimensions are summarized in Figures 3 and 4, respectively.

**TABLE 2:** Summary of penalties of information criteria used in the simulations

Information criterion	Penalty
AIC	2*mc
AICc	$\{2+\frac{2(\mathrm{mc}+1)}{n-\mathrm{mc}-1}\}\times \mathrm{mc}$
BIC	log(n)×mc
mBIC	$\{\log\left(n\right)+2\log\left(\frac{p}{4}-1\right)\}\times \mathrm{mc}$
mBIC2	$\left\{\log\left(n\right)+2\log\left(\frac{p}{4}\right)\right\}\times \mathrm{mc}-2\log\left(\mathrm{mc}!\right)$
GIC	{log(log(n))log(p)}×mc
RIC	2log(p)×mc

FT50 and FT95 denote the *F*‐based method with confidence levels 50% and 95%, respectively. Similarly, GT50 and GT95 are the globaltest‐based method with confidence levels 50% and 95%, respectively. The reason why an alternative significance level 50% is considered is that the traditional methods like CV usually have a type I error rate that is close to 50%. Therefore, if strong type I error rate control is not desired, under similar type I error rate control, the 50% confidence region method becomes comparable to the traditional CV methods.

It can be seen from Figure 3 that the comparisons between the globaltest‐based method and the *F*‐based method is consistent with the result in Table 1. Moreover, it is shown in Figures 3 and 4 that, for the case with positive s where globaltest has good power, GT50 and the CV methods have similar performance in terms of MSE, making GT50 a good alternative to CV but without high computational burden. When s becomes negative, the globaltest‐based method falls behind the other method. However, negative s occurs only seldom in real‐world data sets (Goeman et al., 2006).

For large $r^{2}$ where the variance of the response is largely explained by predictors, AIC in low dimensions and AICc in both low and high dimensions achieve quite good prediction abilities in terms of MSE. This might be due to the small penalties of AIC and AICc compared to the large penalties of BIC, mBIC, mBIC2, GIC, and RIC, which result in underfitting models with an MSE very close to that of the null model, particularly in high dimensions.

Although for extremely small $r^{2}$ where effects of the predictors on the response are extremely weak, globaltest has good power for testing groups of weak effects so that GT50 and GT95 have decent performance on prediction in both low and high dimensions, regardless of the sign of s. Models tuned by BIC, mBIC, mBIC2, GIC, and RIC also predict well in this case because of their large penalties on model complexity, which are mainly dominated by p, especially in high dimensions.

Additionally, we investigate the probability that the null model is chosen when it is true (see Table 3). The confidence region approach can guarantee that the null model is chosen with probability at least 1−α, which is around 95% and 50%, respectively, for GT95 and GT50. We note that the probabilities computed by BIC and its variants and GIC and RIC are large because they adopt a large penalty on model complexity, causing an increasing risk of underfitting models, thereby a high probability that null model is chosen. Although for AICc, it is AIC with an additional penalty on model complexity that is depending on both the sample size and the model complexity itself and can avoid overfitting of AIC to some extent, which is probably the reason that the probability is 0.748 and 0.792 in low and high dimensions in our case.

**TABLE 3:** Probability of choosing λ=∞ when the null model is true

Methods	p=50	p=1000
AIC	0.729	0.000
AICc	0.748	0.792
BIC	0.998	1.000
mBIC	1.000	1.000
mBIC2	1.000	1.000
GIC	1.000	1.000
RIC	1.000	1.000
GCV	0.598	0.644
5‐CV	0.579	0.618
LOOCV	0.583	0.650
GT95	0.953	0.973
GT50	0.508	0.607
FT95	0.947	–
FT50	0.507	–

## 5 REAL DATA EXAMPLES

In the simulation study, we showed the application of confidence region method in linear models. In the real data analysis, we apply the method to Cox models. We consider three high‐dimensional gene expression data sets on breast cancer study: MAINZ with 200 samples and 22,283 gene features (Schmidt et al., 2008); TRANSBIG with 198 samples and 22,283 features (Desmedt et al., 2007); UNT with 137 samples and 44,928 features (Sotiriou et al., 2006). We fit the data by the Cox proportional hazard model with a survival response, which is given by a vector of survival times $t={\left(t_{1},\ldots ,t_{n}\right)}^{T}$ and a vector of status indicators $d={\left(d_{1},\ldots ,d_{n}\right)}^{T}$, where $d_{i}=1$ indicates that $t_{i}$ is an observed survival time and $d_{i}=0$ indicates that the survival time is right‐censored at $t_{i}$. Let $h_{i}\left(t\right)$ denote the hazard function at time t for the ith subject. The Cox proportional hazards model assumes $\log\left(h_{i}\left(t\right)/h_{0}\left(t\right)\right)=x_{i}^{T}\beta$, where $h_{0}\left(t\right)$ is an unspecified underlying hazard. The globaltest confidence region for the Cox model can be obtained by inverting the Cox model version of the globaltest (Goeman et al., 2005).

Cross‐validated partial likelihood (cvpl) can be used as a measure of the predictive ability of Cox models (Verweij & Van Houwelingen, 1993). We compare globaltest‐based method with 5‐fold CV by calculating cvpl, based on 5‐fold CV and 10‐fold CV, respectively. Note that the fold assigning used for estimating the tuning parameter is different from that is used for calculating cvpl. The results are listed in Table 4. The higher the cvpl, the better the predictive performance of the method. It can be seen from Table 4 that there is no large difference of cvpl between CV and confidence region method. CV outperforms the globaltest‐based method in most cases, the globaltest‐based method is, however, much easier to compute than CV without much loss of predictive accuracy.

**TABLE 4:** Cross‐validated partial likelihood by 5‐fold CV and 10‐fold CV

Data sets	Method	5‐fold	10‐fold
MAINZ	CV	−254.90	−257.16
	GT50	−256.71	−259.23
	GT95	−260.11	−262.76
TRANSBIG	CV	−353.71	−358.28
	GT50	−355.90	−359.31
	GT95	−356.66	−359.81
UNT	CV	−149.34	−151.75
	GT50	−149.99	−151.73
	GT95	−150.42	−151.90

**FIGURE 5:**
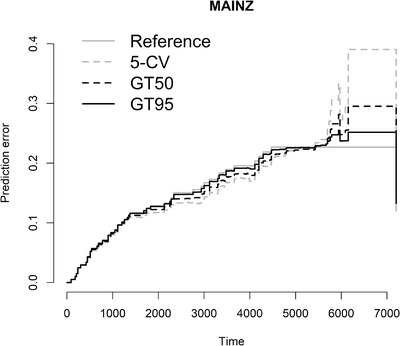
Brier score for MAINZ by the Kaplan–Meier procedure (solid gray line), 5‐CV (dashed gray line), GT50 (dashed black line) and GT95 (solid black line)

**FIGURE 6:**
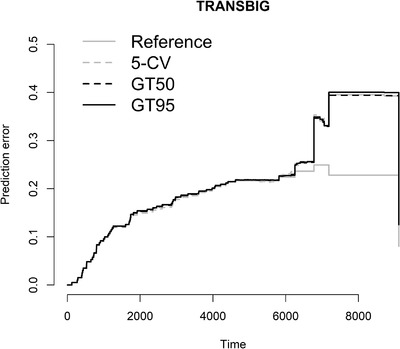
Brier score for TRANSBIG

**FIGURE 7:**
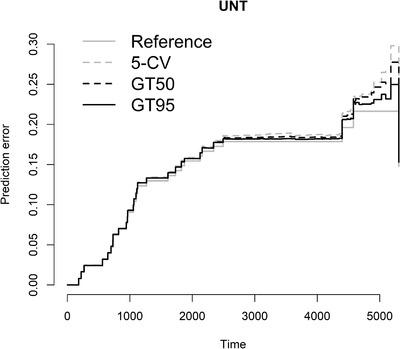
Brier score for UNT

The Brier score is another way to measure the predictive accuracy for survival analysis, which measures the mean squared difference between the predicted survival probability and the actual one (Van Houwelingen & Putter, 2011). It is an overall performance measurement that can be decomposed into two important characteristics of a prediction model, discrimination and calibration (Steyerberg et al., 2010). Figures 5, 6, 7 show the Brier score over time for the models obtained by 5‐CV, GT50, and GT95 in data sets MAINZ, TRANSBIG, and UNT, respectively. The marginal Kaplan–Meier prediction model is presented as a reference to other models. The lower the Brier score, the better the prediction. The results in the figures confirm the conclusion obtained in terms of cvpl that both methods have similar prediction errors, especially at earlier time points. Some differences can be seen at later time points, where the globaltest‐based method predicts the survival probability better than CV, especially for the MAINZ data.

## 6 DISCUSSION

In this work, we constructed the globaltest confidence region, which is powerful to test against high‐dimensional alternatives especially when there are many weak effects, a setting also favorable for ridge regression. We thus proposed to use the globaltest confidence region to choose the tuning parameter of ridge regression, thereby extending the confidence region approach for tuning parameter selection in low to high dimensions by replacing the *F*‐test with the globaltest. We argued that the globaltest has better power than the *F*‐test when strong principal components of the design matrix explain more variance of the outcome than the weak ones, which is common in real‐world applications.

The tuning parameter selected by the globaltest confidence region is the parameter corresponding to the first time that the ridge path reaches the boundary of the confidence region at a specified level α, or is the infinity when the whole path included in the region. It can be seen as the least complex model among all acceptable models. Tuning via confidence regions is computationally less demanding than CV. Compared with information criteria, the confidence region method has less dependence on the penalties on the model complexity. And, as a testimation procedure, it further guarantees that the null model is selected with a prespecified probability in the case that it is the true model. This can be linked to the weak family‐wise error rate control from the perspective of multiple testing.

An important asset of the globaltest‐based method is that it is known when this method is expected to perform well, that is, when the strong principal components dominate signals or when there are many weak signals. We focused on ridge regression because it is similar in spirit to the globaltest, but in principle our approach may be used for other penalized method for model selection as well. With regard to multiple testing corrections applied to model selection, such as family‐wise error rate and false discovery rate, see Żak‐Szatkowska and Bogdan (2011) for more detail.

## CONFLICT OF INTEREST

The authors have declared that there is no conflict of interest.

### OPEN RESEARCH BADGES

This article has earned an Open Data badge for making publicly available the digitally‐shareable data necessary to reproduce the reported results. The data is available in the Supporting Information section.

This article has earned an open data badge “**Reproducible Research**” for making publicly available the code necessary to reproduce the reported results. The results reported in this article could fully be reproduced.

## Supporting information

Supporting R functions and source code to reproduce the results are available from the author or on the journal's web page https://doi.org/10.1002/bimj.202000063

## Data Availability

The data that support the findings of this study are openly available in bioconductor with doi: https://doi.org/10.18129/B9.BIOC.BREASTCANCERMAINZ, https://doi.org/10.18129/B9.BIOC.BREASTCANCERTRANSBIG, https://doi.org/10.18129/B9.BIOC.BREASTCANCERUNT.
