# Supplementary figures and images for: Globaltest confidence regions and their application to ridge regression
Source: Biom J. 2021 May 27;63(7):1351–65. doi: 10.1002/bimj.202000063 (PMC8519024; doi:10.1002/bimj.202000063)

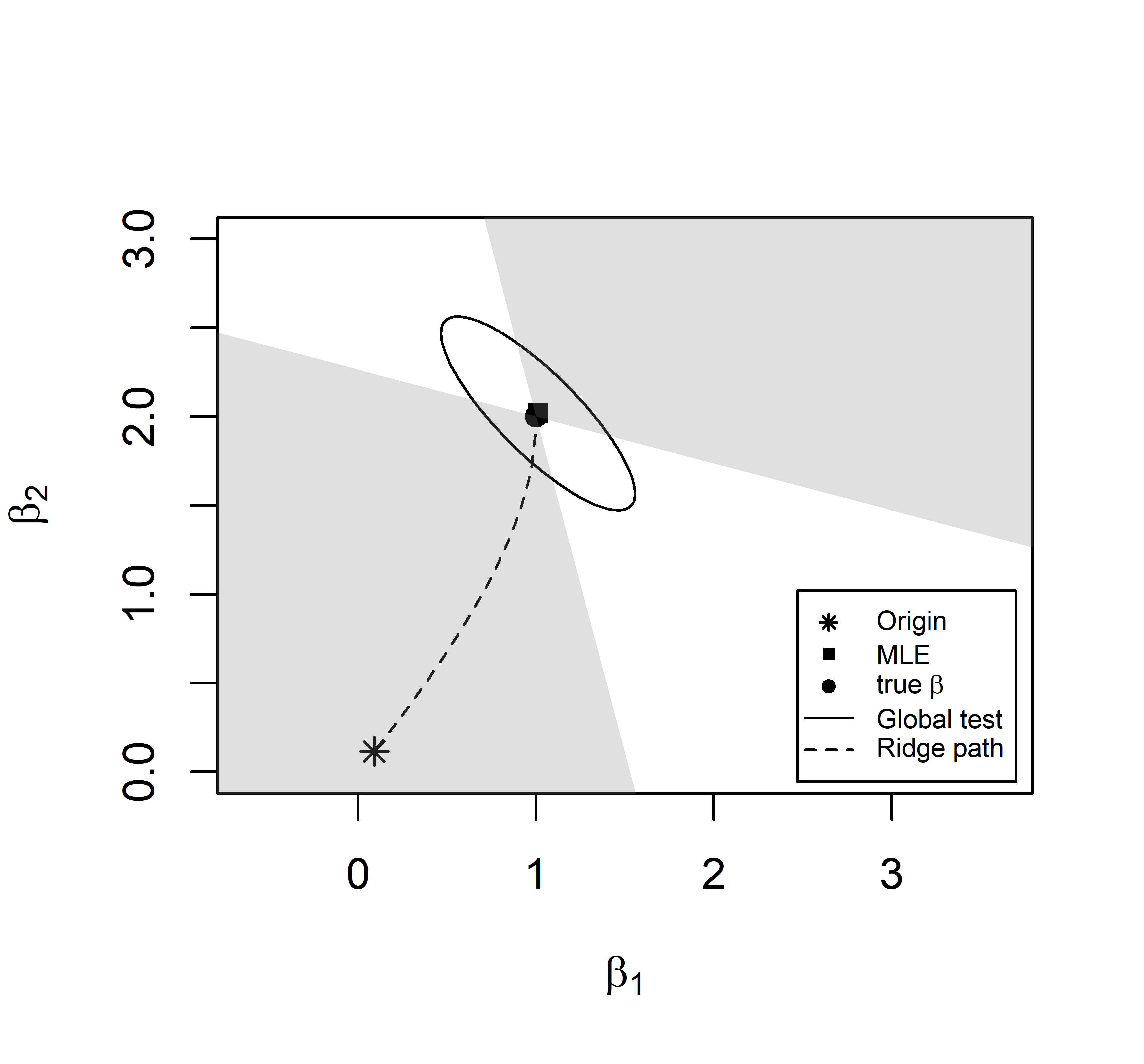

Supplement: Supplementary file 1 — Supporting R functions and source code to reproduce the results are available from the author or on the journal's web page https://doi.org/10.1002/bimj.202000063 [file BIMJ-63-1351-s001.zip › code_and_data/confidence-region-example/Fig1_detect.png]

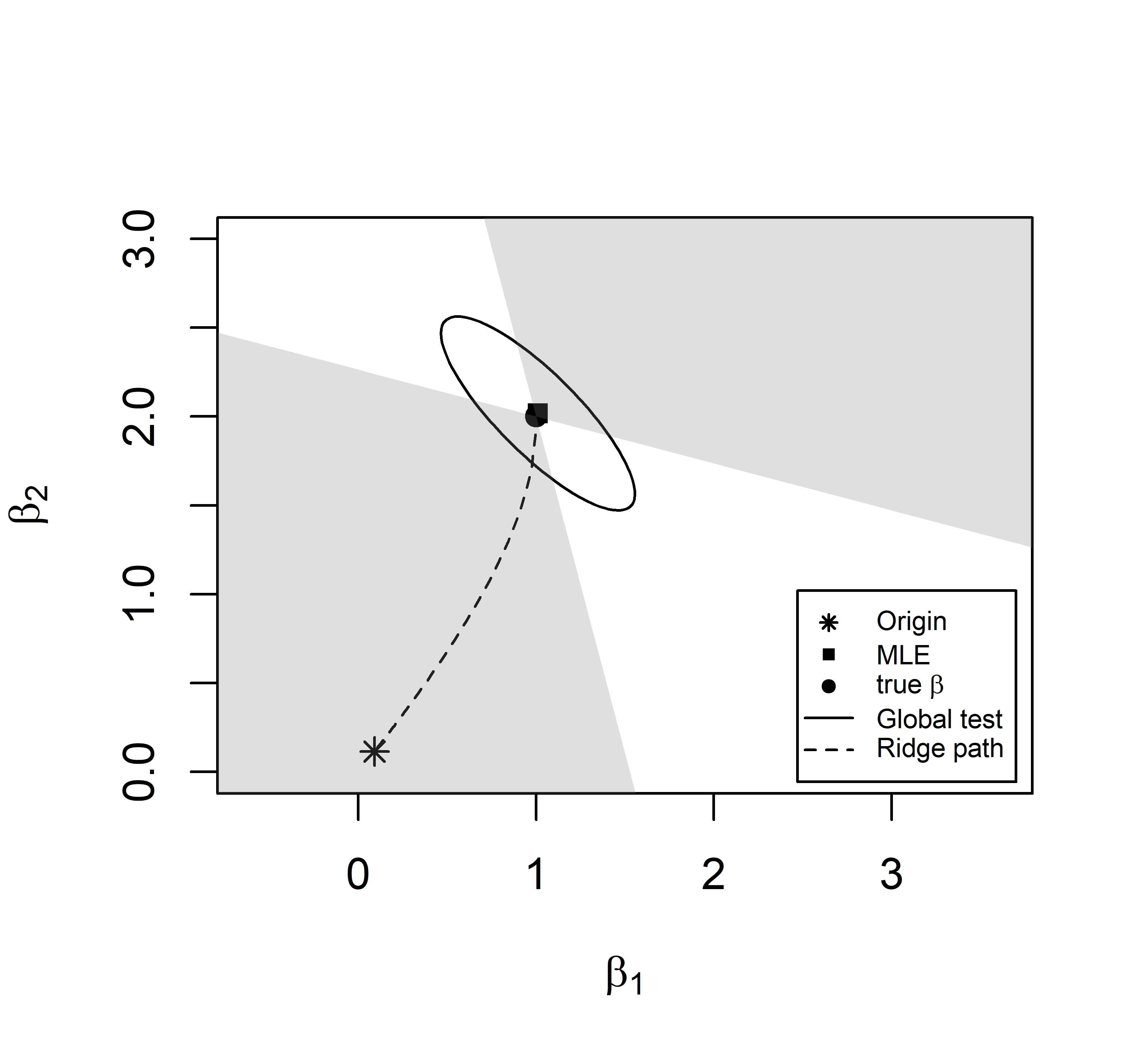

Supplement: Supplementary file 1 — Supporting R functions and source code to reproduce the results are available from the author or on the journal's web page https://doi.org/10.1002/bimj.202000063 [file BIMJ-63-1351-s001.zip › code_and_data/confidence-region-example/Fig1_detect.tiff]

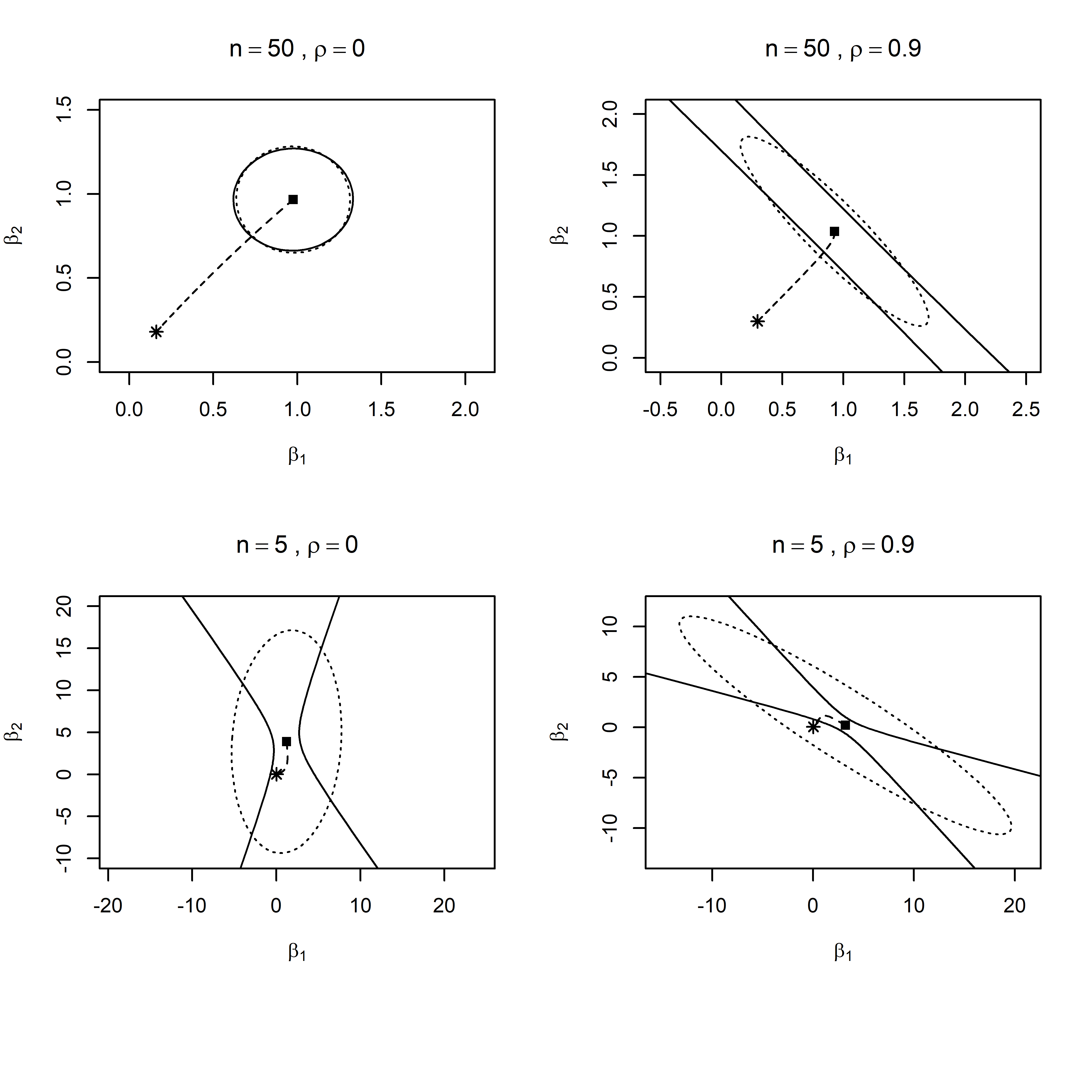

Supplement: Supplementary file 1 — Supporting R functions and source code to reproduce the results are available from the author or on the journal's web page https://doi.org/10.1002/bimj.202000063 [file BIMJ-63-1351-s001.zip › code_and_data/confidence-region-example/Fig2_gtft.png]

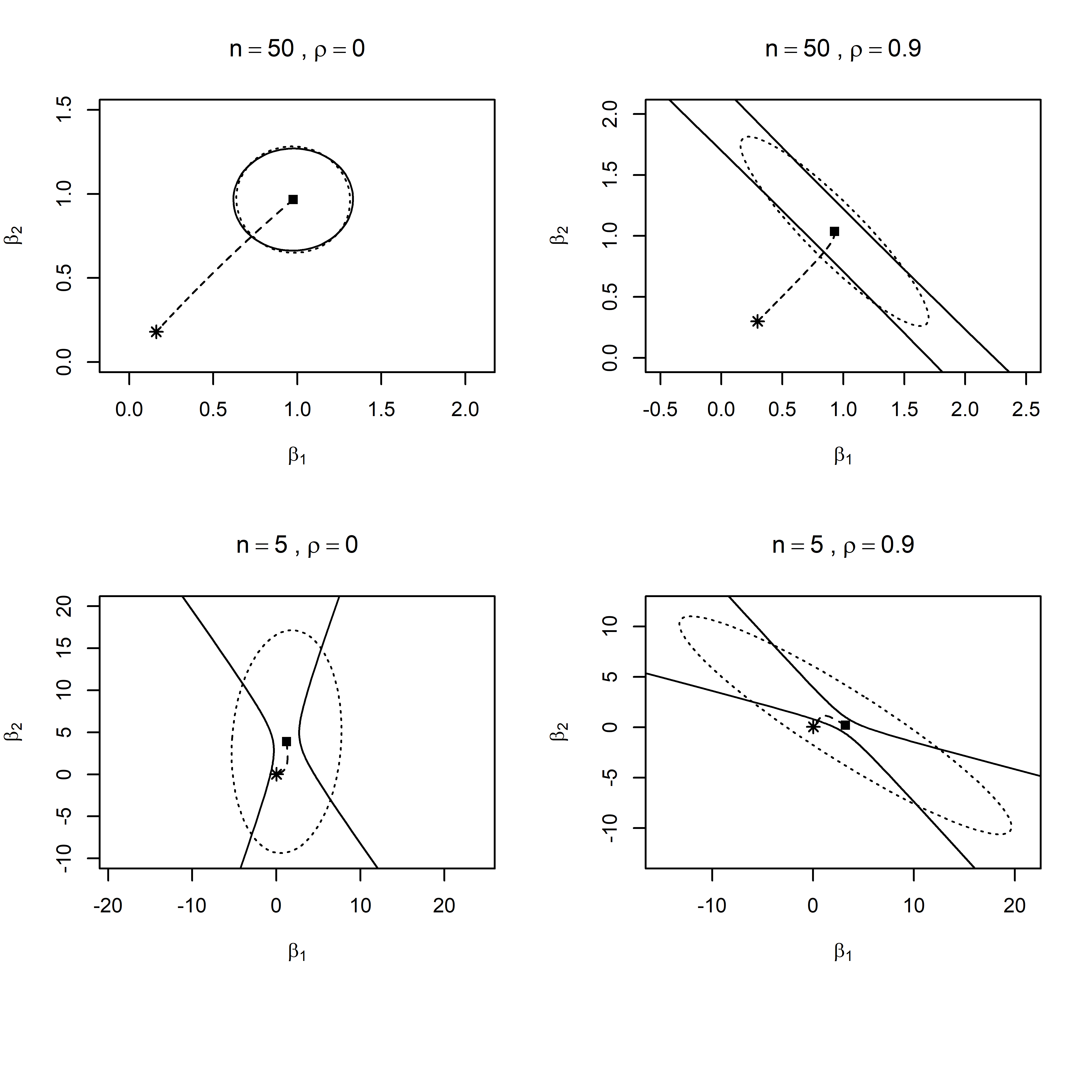

Supplement: Supplementary file 1 — Supporting R functions and source code to reproduce the results are available from the author or on the journal's web page https://doi.org/10.1002/bimj.202000063 [file BIMJ-63-1351-s001.zip › code_and_data/confidence-region-example/Fig2_gtft.tiff]

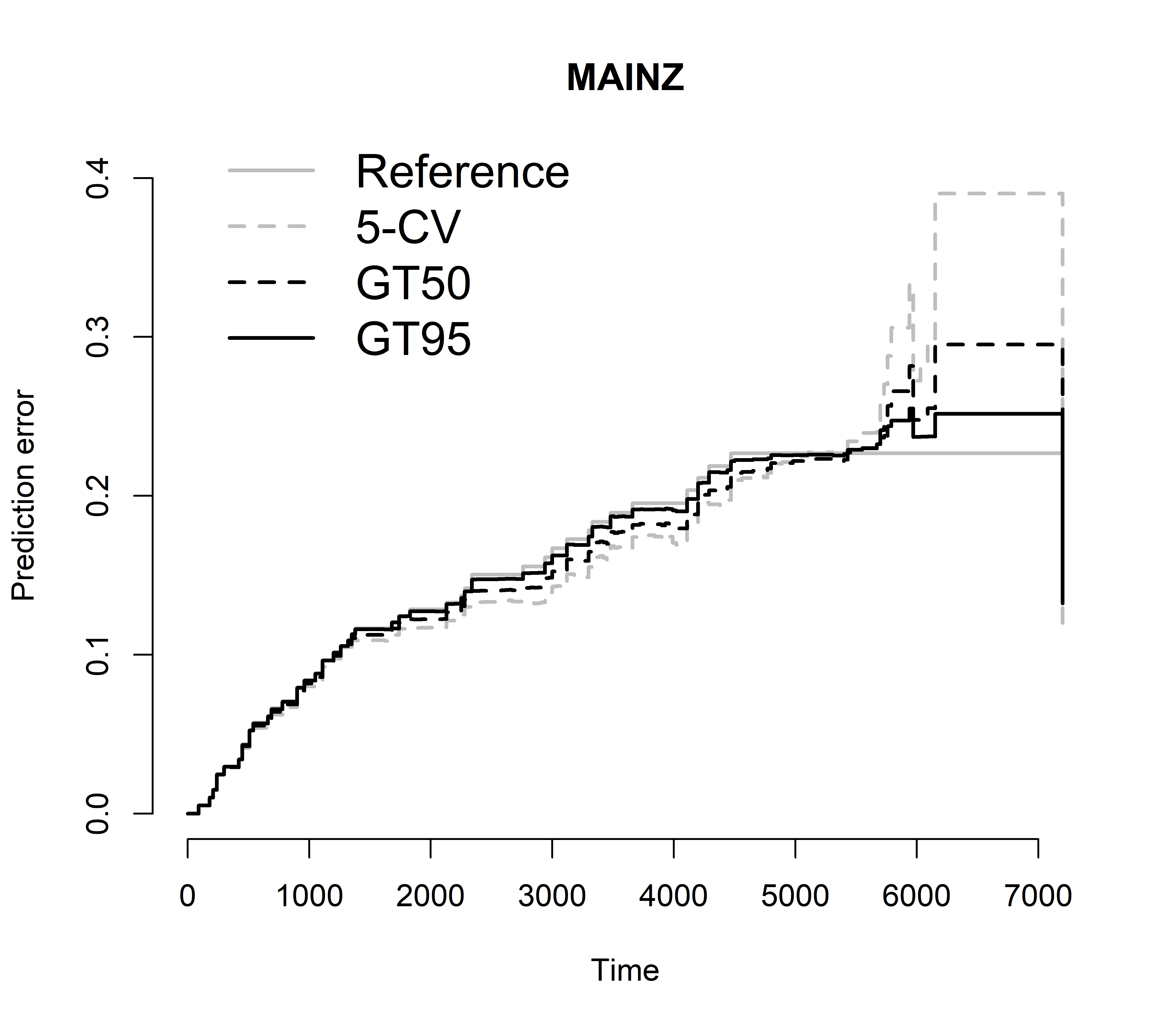

Supplement: Supplementary file 1 — Supporting R functions and source code to reproduce the results are available from the author or on the journal's web page https://doi.org/10.1002/bimj.202000063 [file BIMJ-63-1351-s001.zip › code_and_data/realdata/brierscore/Fig5_bsm.png]

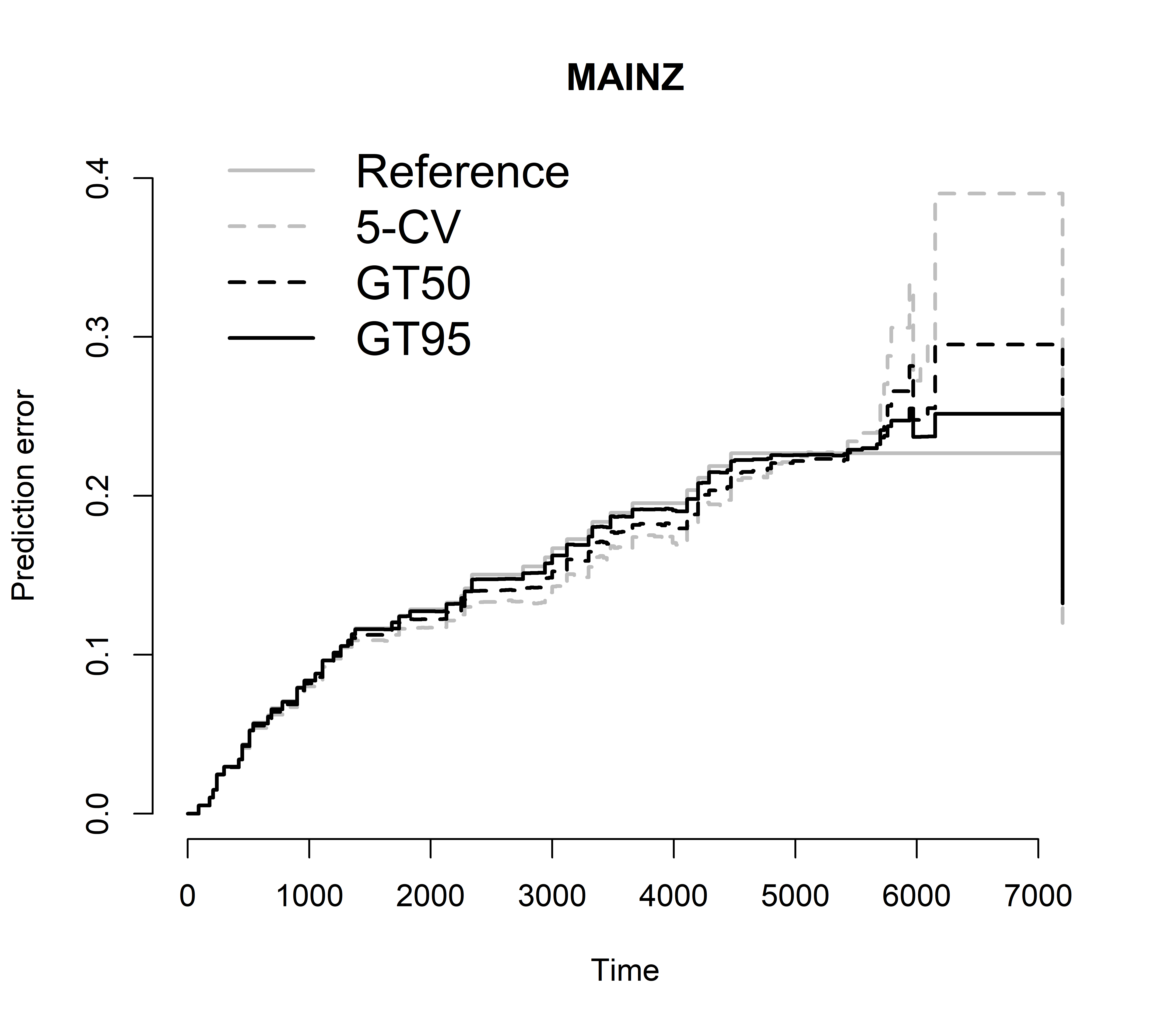

Supplement: Supplementary file 1 — Supporting R functions and source code to reproduce the results are available from the author or on the journal's web page https://doi.org/10.1002/bimj.202000063 [file BIMJ-63-1351-s001.zip › code_and_data/realdata/brierscore/Fig5_bsm.tiff]

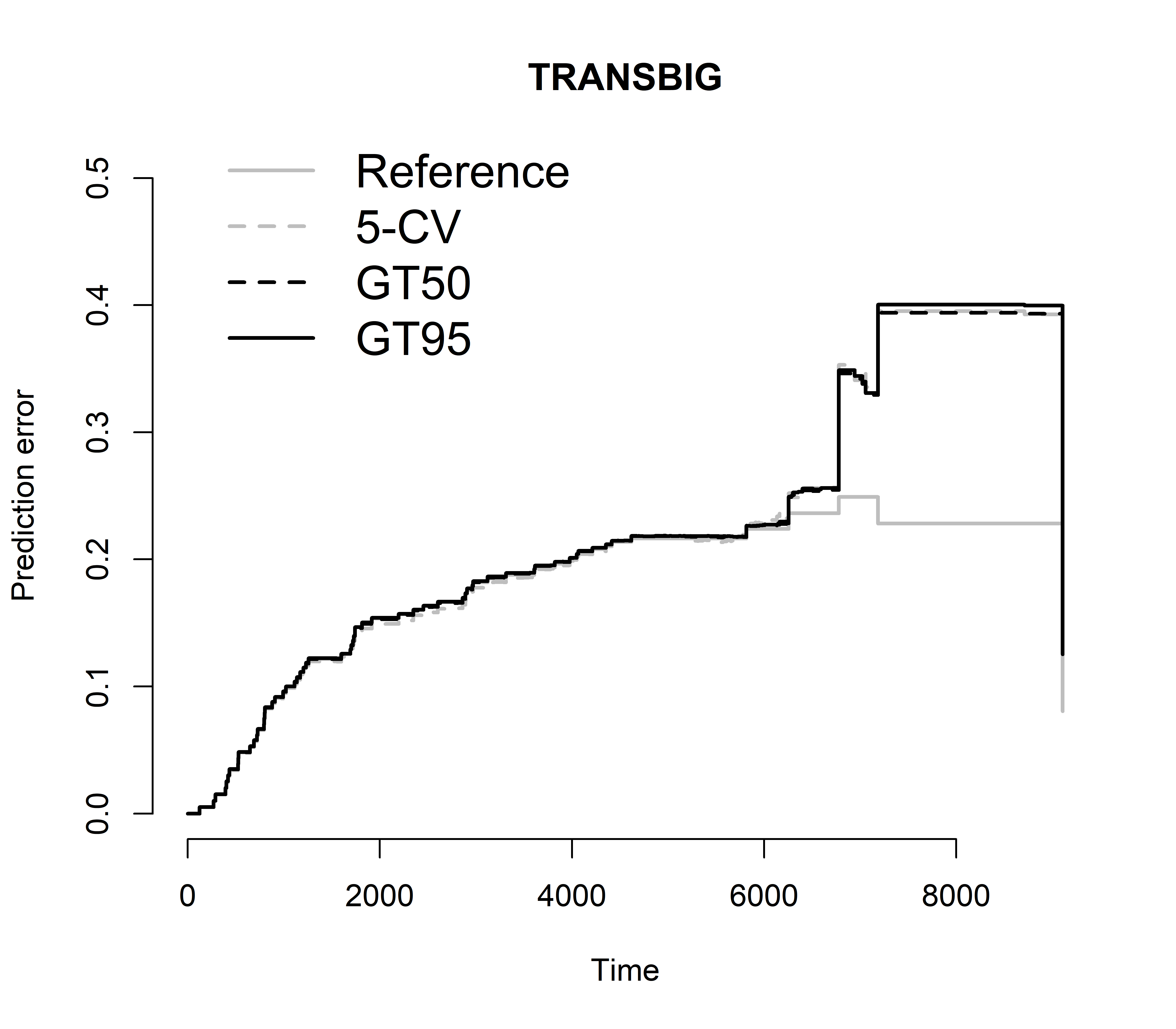

Supplement: Supplementary file 1 — Supporting R functions and source code to reproduce the results are available from the author or on the journal's web page https://doi.org/10.1002/bimj.202000063 [file BIMJ-63-1351-s001.zip › code_and_data/realdata/brierscore/Fig6_bst.png]

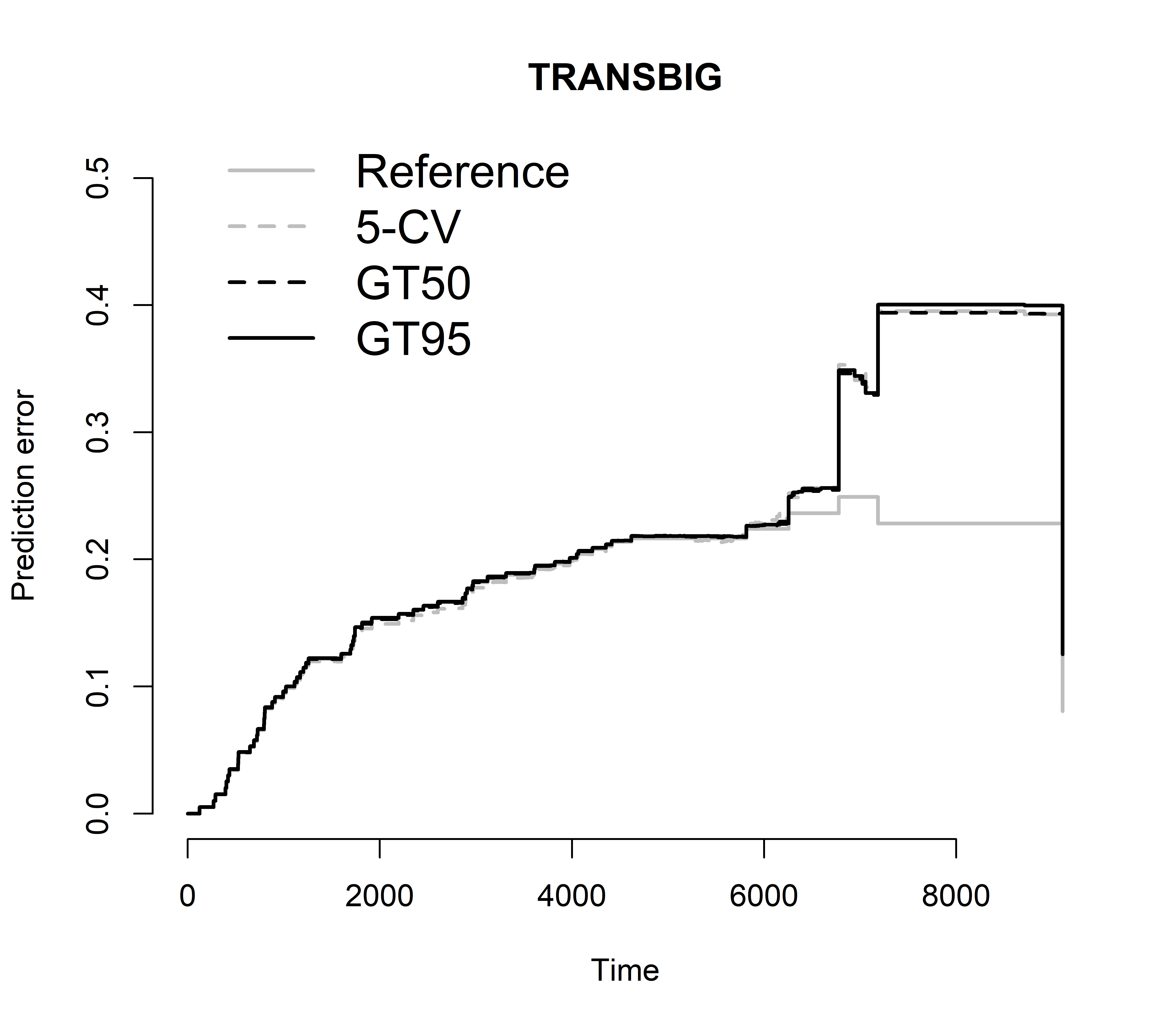

Supplement: Supplementary file 1 — Supporting R functions and source code to reproduce the results are available from the author or on the journal's web page https://doi.org/10.1002/bimj.202000063 [file BIMJ-63-1351-s001.zip › code_and_data/realdata/brierscore/Fig6_bst.tiff]

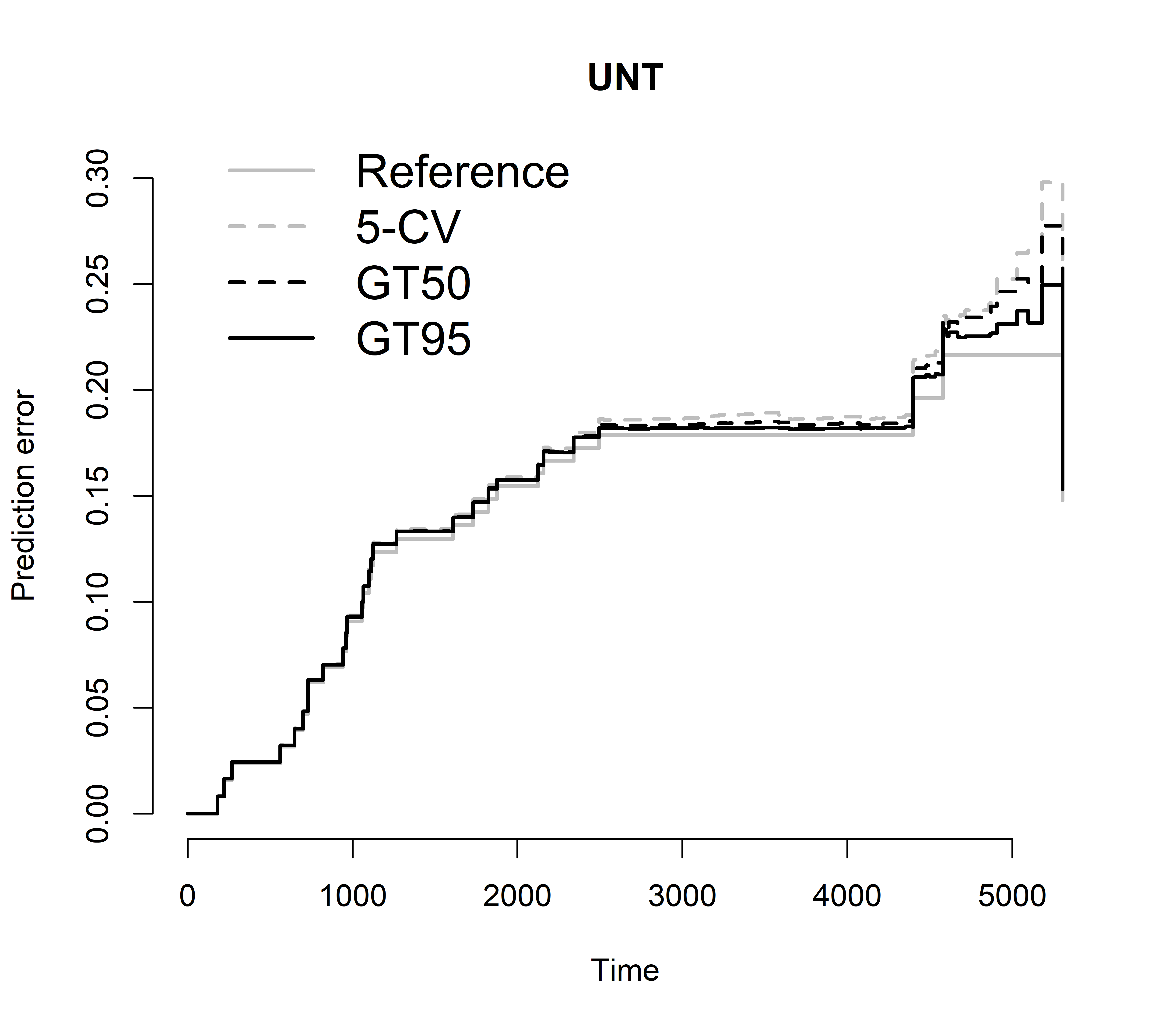

Supplement: Supplementary file 1 — Supporting R functions and source code to reproduce the results are available from the author or on the journal's web page https://doi.org/10.1002/bimj.202000063 [file BIMJ-63-1351-s001.zip › code_and_data/realdata/brierscore/Fig7_bsu.png]

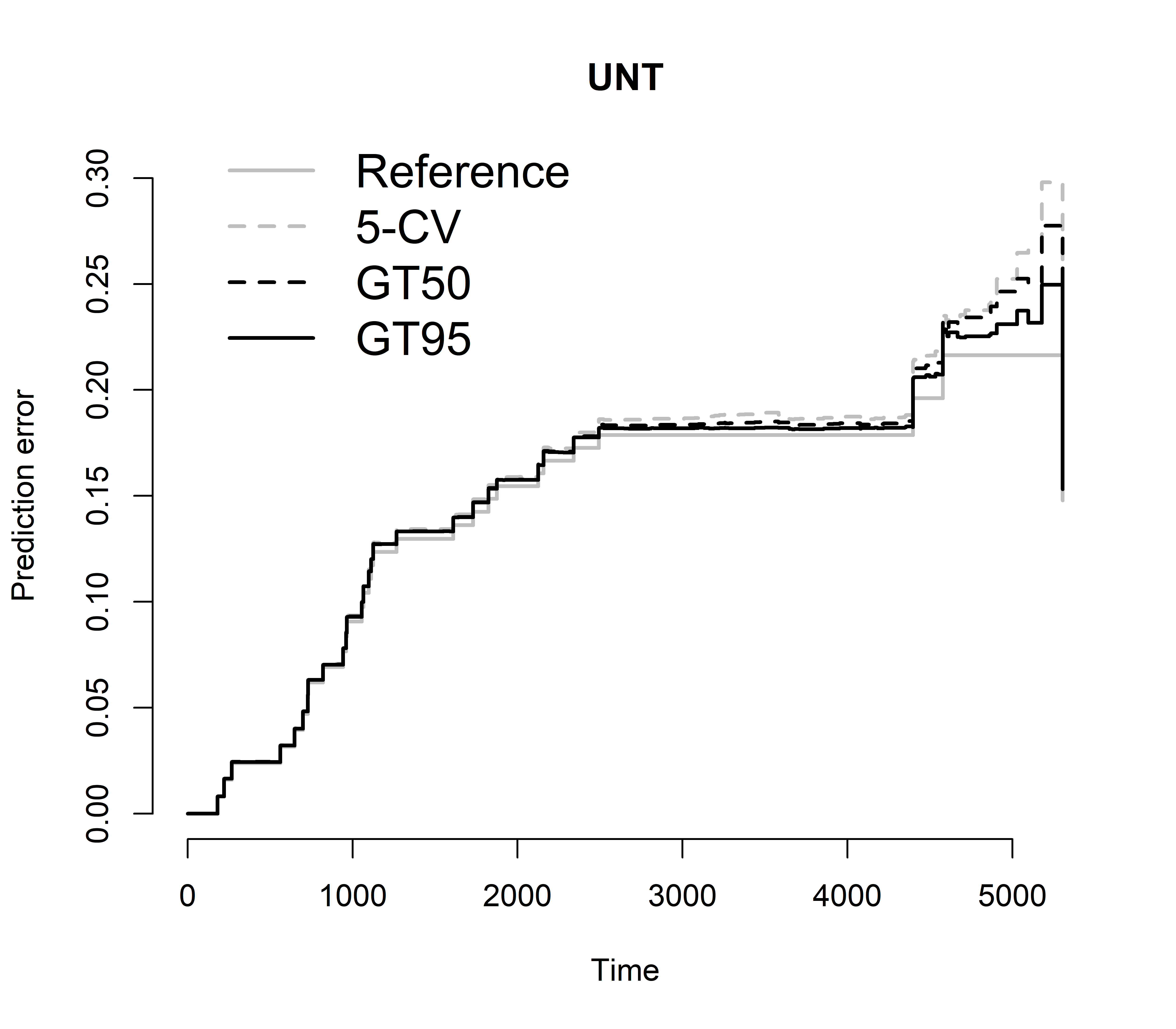

Supplement: Supplementary file 1 — Supporting R functions and source code to reproduce the results are available from the author or on the journal's web page https://doi.org/10.1002/bimj.202000063 [file BIMJ-63-1351-s001.zip › code_and_data/realdata/brierscore/Fig7_bsu.tiff]

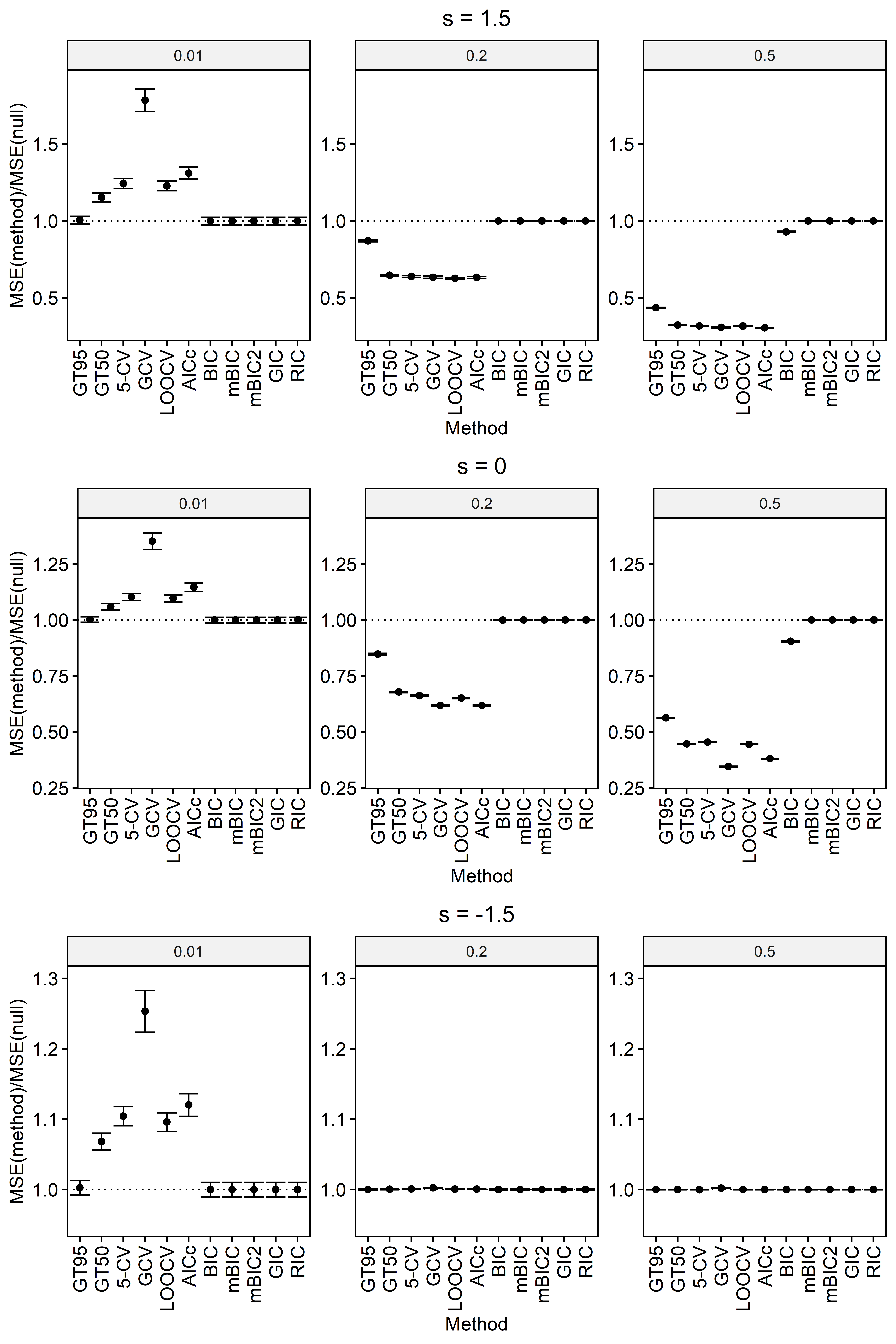

Supplement: Supplementary file 1 — Supporting R functions and source code to reproduce the results are available from the author or on the journal's web page https://doi.org/10.1002/bimj.202000063 [file BIMJ-63-1351-s001.zip › code_and_data/simulation/mse/high/Fig4.png]

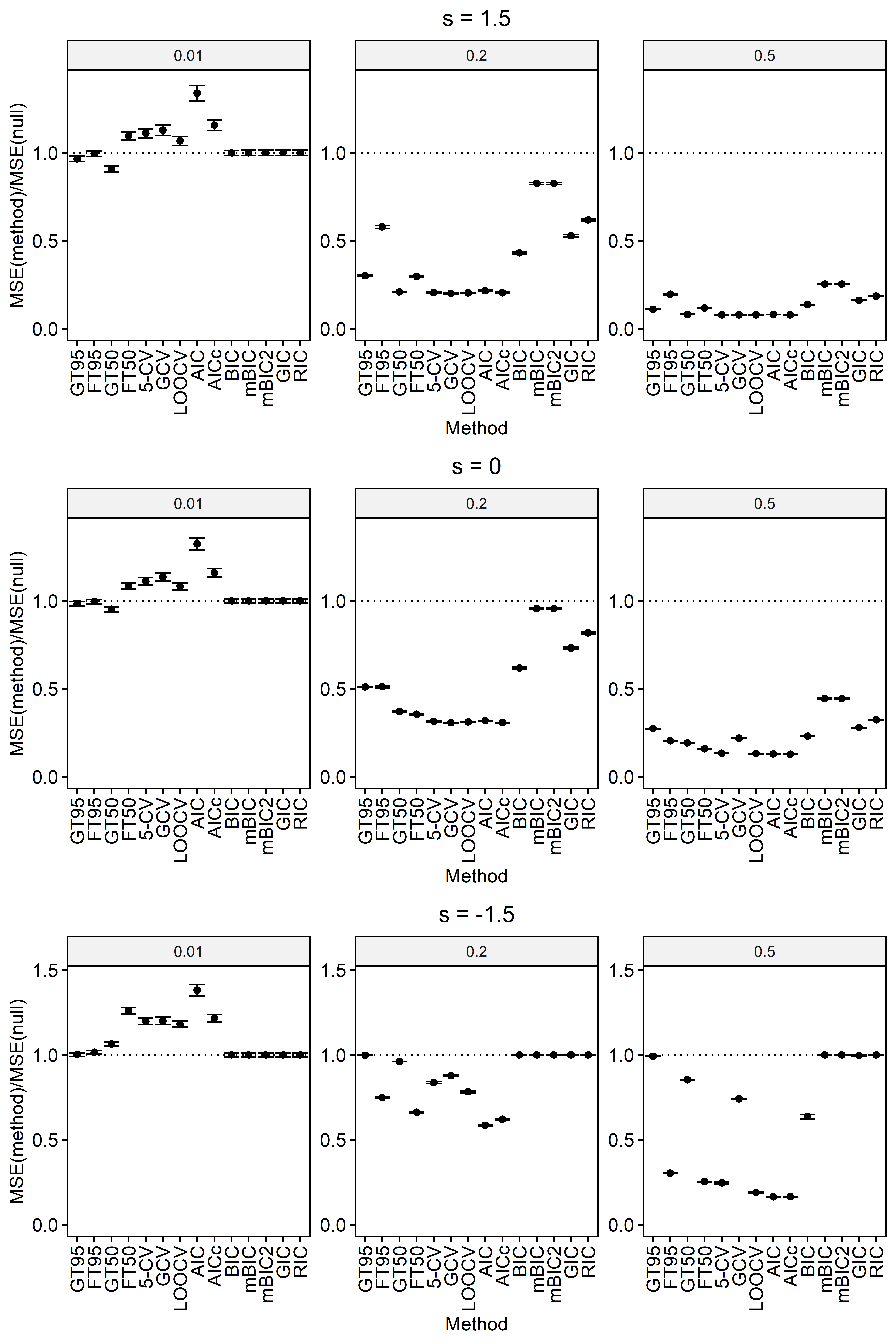

Supplement: Supplementary file 1 — Supporting R functions and source code to reproduce the results are available from the author or on the journal's web page https://doi.org/10.1002/bimj.202000063 [file BIMJ-63-1351-s001.zip › code_and_data/simulation/mse/low/Fig3.png]
